# Human Choice Predicted by Obtained Reinforcers, Not by Reinforcement Predictors

**DOI:** 10.3389/fpsyg.2020.01631

**Published:** 2020-07-24

**Authors:** Jessica P. Stagner, Vincent M. Edwards, Sara R. Bond, Jeremy A. Jasmer, Robert A. Southern, Kent D. Bodily

**Affiliations:** Department of Psychology, Georgia Southern University, Statesboro, GA, United States

**Keywords:** suboptimal choice, choice, matching law, preference, comparative psychology

## Abstract

Macphail (1985) proposed that “intelligence” should not vary across vertebrate species when contextual variables are accounted for. Focusing on research involving choice behavior, the propensity for choosing an option that produces stimuli that predict the presence or absence of reinforcement but that also results in less food over time can be examined. This choice preference has been found multiple times in pigeons (Stagner and Zentall, 2010; Zentall and Stagner, 2011; Laude et al., 2014) and has been likened to gambling behavior demonstrated by humans (Zentall, 2014, 2016). The present experiments used a similarly structured task to examine adult human preferences for reinforcement predictors and compared findings to choice behavior demonstrated by children (Lalli et al., 2000), monkeys (Smith et al., 2017; Smith and Beran, 2020), dogs (Jackson et al., 2020), rats (Chow et al., 2017; Cunningham and Shahan, 2019; Jackson et al., 2020), and pigeons (Roper and Zentall, 1999; Stagner and Zentall, 2010). In Experiment 1, adult human participants showed no preference for reinforcement predictors. Results from Experiment 2 suggest that not only were reinforcement predictors not preferred, but that perhaps reinforcement predictors had no effect at all on choice behavior. Results from Experiments 1 and 2 were further assessed using a generalized matching equation, the findings from which support that adult human choice behavior in the present research was largely determined by reinforcement history. Overall, the present results obtained from human adult participants are different than those found from pigeons in particular, suggesting that further examination of Macphail (1985) hypothesis is warranted.

## Introduction

Macphail (1985) argued that comparative psychologists should adopt the assumption of general processes of learning. That is, despite the common notion that learning capacities vary between species and that species may be ranked by these capacities, the null hypothesis in the comparison of behavioral traits across species must be that there are no differences. Macphail specified that research had found no cross-species differences with regard to either qualitative (i.e., differences in mechanism) or quantitative (i.e., differences in efficiency of a shared mechanism). According to Macphail, the failure to convincingly rule out the null hypothesis was due to an absence of systematic replications to rule out contextual variables (e.g., motivating operations, stimulus characteristics, etc.). Parsimony, Macphail stated, requires any apparent differences must first be ascribed to contextual variables. Macphail’s only exception was that verbal humans were to be excluded from this argument. But what if humans were tested in procedures that were analogous to those applied to non-humans? Here we reviewed selected systematic replications of choice behavior in human and non-human animals and presented two experiments with human participants to test Macphail’s Null Hypothesis, which stated that there should be no cross-species differences in general learning processes.

**FIGURE 1:**
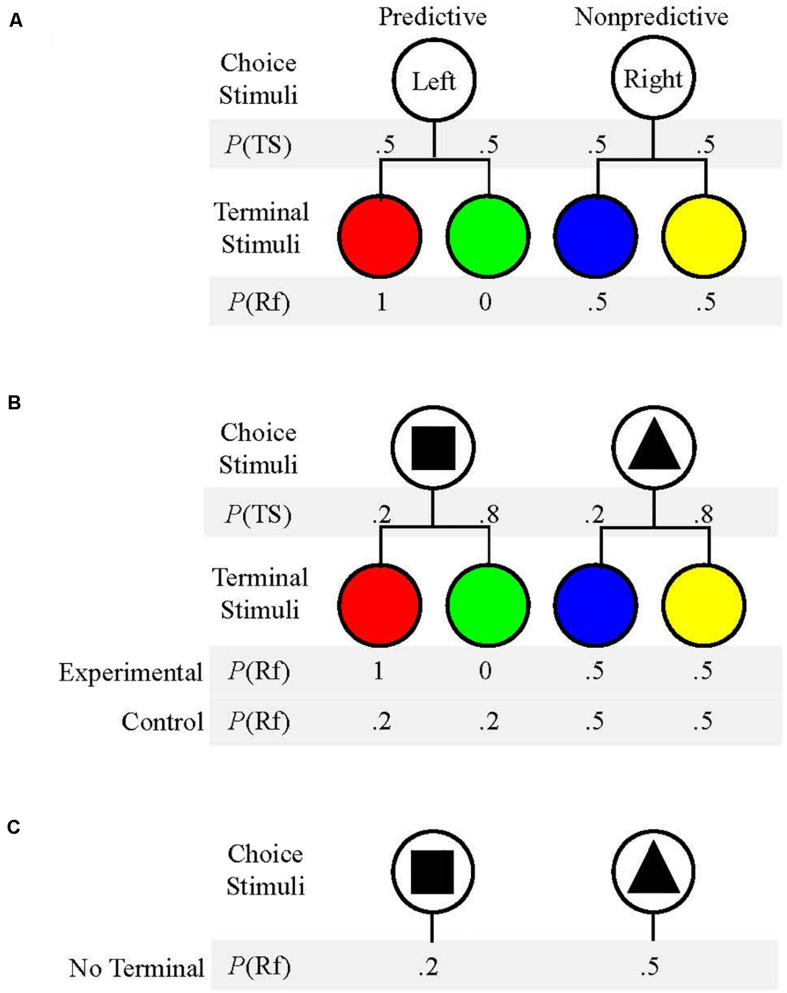
**(A)** Schematic of the procedure used by Roper and Zentall (1999). Pigeons chose between the Left and Right keys (Choice Stimuli). *P*(TS) is the probability that each Terminal Stimulus would occur following a response. *P*(Rf) is the probability that reinforcement would follow each Terminal Stimulus. **(B)** Schematic of the procedure used by Stagner and Zentall (2010). Choice Stimuli were shapes presented on left or right sides with equal likelihood. The probability of each Terminal Stimulus, *P*(TS), and probability of reinforcement following each Terminal Stimulus, *P*(Rf), are given for each condition. **(C)** Schematic of procedure used in Experiment 2. Choice Stimuli were presented without any Terminal Stimuli. P(Rf) is the probability that reinforcement followed each Choice Stimulus.

Past research has demonstrated preference for an alternative which produces stimuli that signal the future presence or absence of reinforcement over an alternative which does not produce reinforcement-predictive stimuli. This preference has been found in multiple species, including capuchins, rhesus macaques, pigeons, and rats (Roper and Zentall, 1999; Smith et al., 2017; Cunningham and Shahan, 2019; Smith and Beran, 2020). For example, when Roper and Zentall (1999) presented a choice between two alternatives which produced reinforcement equally 50% of the time, pigeons’ choices were more frequently allocated to the alternative which provided reinforcement-predictive stimuli (see Figure 1A). This preference is interesting in that pigeons did not obtain any additional food by choosing the predictive alternative. Additionally, the preference for predictive stimuli increased when the likelihood of food reinforcement for each alternative was reduced from 50% to only 12.5%. Thus, it appears that predictive stimuli might have been more valued when overall probability of reinforcement was low (Roper and Zentall, 1999).

To test the strength of the preference for predictive stimuli, Stagner and Zentall (2010) used a similar procedure, but the two choice options did not payout equally. The “suboptimal” alternative produced predictive stimuli but only resulted in reinforcement 20% of the time whereas the “optimal” alternative was not followed by predictive stimuli but resulted in reinforcement 50% of the time (see Figure 1B). If pigeons’ choices were determined by obtained reinforcement, they should have shown a clear preference for the “optimal” option that provided two and half times more food. However, if predictors of reinforcement were valued, particularly when reinforcement was somewhat scarce due to the lean schedule, then pigeons should have shown a preference for the “suboptimal” alternative that resulted in predictive stimuli but paid out less. Stagner and Zentall (2010) found that pigeons demonstrated a near exclusive preference for the “suboptimal” option that provided predictive stimuli despite the fact that it yielded much less food over time. Pigeons were also given the same task, but choosing both the “optimal” alternative and the “suboptimal” alternative produced non-predictive stimuli. In this case, pigeons chose the “optimal” alternative that yielded more food (50%). Thus, it seems that the predictive nature of the stimuli used in this task was the mechanism for the “suboptimal” preference found by pigeons (Stagner and Zentall, 2010).

Considering these findings (Stagner and Zentall, 2010), the preference found for a “suboptimal” alternative was not due to pigeons’ inability to detect the difference in yield between the two alternatives. Rather, this preference provides more support that reinforcement predictors were valued such that pigeons were foregoing food to choose the option that provided them. This finding has been suggested to be analogous to human gambling behavior in that humans that gamble incur losses but continue to engage in the behavior (Stagner and Zentall, 2010; Zentall, 2014; Zentall, 2016).

Rats have also been tested for a preference for predictive stimuli using a two lever task in which the “suboptimal” lever produced predictive stimuli but less food over time, while the “optimal” lever resulted in more food over time but no reinforcement predictors (Cunningham and Shahan, 2019). Interestingly, few rats preferred the “suboptimal” alternative that produced predictive stimuli when those stimuli were presented for the duration of 10 s that has been effective in many previous studies (Stagner and Zentall, 2010; Zentall and Stagner, 2011; Stagner et al., 2012; Chow et al., 2017). Moreover, the duration for these predictive stimuli had to be extended to at least 30 s before eight of the nine rats in the task preferred the “suboptimal” predictive alternative relative to chance (Cunningham and Shahan, 2019). A similar study also done with rats found a preference for predictive stimuli despite that it resulted in half as much food over time when compared to its non-predictive counterpart (Chow et al., 2017). Within this study, two initial alternatives required nose-poke responses to indicate a choice selection. Following selection of the “suboptimal” alternative that produced predictive stimuli, terminal predictive stimuli were either a 10 s light presentation or a 10 s lever presentation to signal reinforcement while a 10 s blackout was used to signal no reinforcement. Rats that received a lever as a terminal stimulus signaling reinforcement chose the predictive suboptimal alternative whereas rats that received a light signaling reinforcement did not (Chow et al., 2017). However, when rats were tested with this two-alternative task but with odors used as predictive terminal stimuli, rats showed no preference for reinforcement predictors (Jackson et al., 2020). Taken together, these studies demonstrate that under certain conditions (i.e., contextual variables) rats may prefer predictive stimuli, even if it comes at the cost of food resources. It is important to note that between the two of these studies that found evidence of a preference for predictive stimuli by rats (Chow et al., 2017; Cunningham and Shahan, 2019), neither found the same degree of preference for predictive stimuli that has been consistently found by pigeons (Stagner and Zentall, 2010; Zentall and Stagner, 2011; Stagner et al., 2012; Laude et al., 2014). That is, despite the systematic variations of contextual variables, these results may demonstrate a quantitative difference in preference for reinforcement predictors between rats and pigeons, which should not occur according to Macphail (1985).

When a similar study was conducted with rhesus macaques, subjects were more likely to choose a risky option which gave reinforcement less often if the outcomes were signaled (Smith et al., 2017). That is, with experience, macaques chose suboptimally more often if stimuli predictive of reinforcement outcomes followed those choices. Interestingly, macaques chose suboptimally around 64% of the time after several sessions of experience (Smith et al., 2017). This finding suggests a quantitative difference when comparing the preference for predictive stimuli by macaques (Smith et al., 2017) to the preference for predictive stimuli by pigeons (Stagner and Zentall, 2010; Zentall and Stagner, 2011; Stagner et al., 2012; Laude et al., 2014).

Human participants have also been tested with similar procedures to assess preference for reinforcement predictive stimuli. Lalli et al. (2000) used a two-alternative task to assess if children with developmental delays would demonstrate a preference for reinforcement predictors. Children were given an initial choice between two black boxes. Choice of the optimal box always produced a colored block followed by reinforcement after a 30 s terminal duration. Choice of the suboptimal box produced either a 30 s colored block that signaled reinforcement or a 30 s colored block that was predictive of non-reinforcement. Lalli et al. (2000) found that children chose the box that resulted in less reinforcement as long as the colored blocks predicted the reinforcement outcome. In a condition in which the colored blocks were not predictive of reinforcement, children began to choose the optimal box option. Interestingly, when 10 s terminal durations were used for the colored block stimuli, children chose optimally. In a second experiment replicating a pigeon procedure that inserted a 10 s delay between the choice of box and the presentation of the 30 s colored block stimulus duration (Belke and Spetch, 1994), children chose the optimal box alternative that provided reinforcement 100% of the time (Lalli et al., 2000).

Taken together, these findings support that children with developmental delays, like pigeons, rats, and macaques, will choose suboptimally, under certain conditions, if stimuli are provided that predict reinforcement outcomes. However, it is important to note that children (Lalli et al., 2000) did not have the same strength in preference for predictive stimuli that has been consistently found with pigeons (Stagner and Zentall, 2010; Zentall and Stagner, 2011; Stagner et al., 2012; Laude et al., 2014). Thus, there appears to be more similarity in choice allocation between developmentally delayed children (Lalli et al., 2000) and rhesus macaques (Smith et al., 2017) within this two-alternative task, while a quantitative difference seems to be present when comparing children and macaques to pigeons.

Further exploration employed adult human participants and aimed to observe how preference for predictive outcomes might influence human gambling behavior. A similar two-alternative task was used but was presented to adult human participants in the format of a video game (Molet et al., 2012). During choice trials, participants were allowed to select one of two planetary systems to kill as many generals as possible. Choosing the “suboptimal” system produced stimuli which predicted the number of generals that would be killed but resulted in fewer generals killed over time. Choosing the “optimal” system produced non-predictive stimuli but resulted in more generals killed over time. Participants in this study were selected based on their responses to a survey they completed in a screening during an introductory psychology course. Specifically, participants that reported that they engaged in gambling behaviors were assigned to the “gambling habit” group, whereas those that reported no such engagement were assigned to the “non-gambling habit” group. Participants in the “gambling habit” group selected the “suboptimal” planetary system 56.5% of the time on average, whereas participants in the “non-gambling habit” group selected the “suboptimal” system only 23% of the time on average. This was taken as support that self-reported gamblers made more “suboptimal” choices. It is important to note that while self-reported gamblers chose less optimally than self-reported non-gamblers in this task, neither group chose the “suboptimal” predictive alternative as often as has been demonstrated by pigeons (Stagner and Zentall, 2010; Zentall and Stagner, 2011).

Recently, McDevitt et al. (2019) attempted to replicate previous findings in pigeons and adult humans using a two-alternative task. While pigeons performed similarly to past studies in that they preferred a suboptimal alternative that produced reinforcement-predictive stimuli, adult human participants showed no such preference (McDevitt et al., 2019). Specifically, adult human participants demonstrated a clear preference for an optimal alternative that did not produce predictive stimuli. When an unsignaled condition was employed in which neither alternative produced predictive stimuli, adult human participants also chose the optimal alternative (McDevitt et al., 2019). Interestingly, these results are similar to those found by Molet et al. (2012) with adult human participants, but contrasts with results from developmentally delayed children (Lalli et al., 2000), rhesus macaques (Smith et al., 2017), rats (Chow et al., 2017; Cunningham and Shahan, 2019), and pigeons (Stagner and Zentall, 2010; Zentall and Stagner, 2011). Collectively, within the range of contextual variables that have been systematically investigated, it seems that there is a difference between vertebrate species when observing preference for reinforcement-predictive stimuli. This finding contrasts with the notion that these phenomena would not differ in nature across vertebrate species (Macphail, 1985).

The present experiments were conducted to continue to explore the preference for predictive stimuli in adult human participants. In Experiment 1, participants were presented with a computer task that replicated the method of Stagner and Zentall (2010, see Figure 1B). To assess if terminal stimulus duration would affect choice behavior as has been found with rats (Cunningham and Shahan, 2019), the terminal stimulus duration was systematically manipulated across three conditions: 2, 8, and 20 s terminal durations. This procedure was chosen to allow for direct comparison to other studies conducted with pigeons (Stagner and Zentall, 2010; Zentall and Stagner, 2011) and with rats (Chow et al., 2017; Cunningham and Shahan, 2019). If human participants prefer predictive stimuli as pigeons, and sometimes rats, do within this task structure, then participants should select the alternative that provides those stimuli but that pays out less often. This finding would also support the hypothesis that this preference/susceptibility is the same across vertebrate species (Macphail, 1985). Alternatively, if participants’ choices do not correspond to predictive stimuli, it might suggest that there is a difference between these species.

## Experiment 1

### Method

#### Participants

Participants (*N* = 73) were undergraduate students over the age of 18 who were recruited from a subject pool of the Department of Psychology at Georgia Southern University. They selected to participate using SONA Systems^1^ and received course credit for their participation. Participants signed a consent form before beginning an experimental session. Once the experimental session was completed, participants were debriefed. No deception was used in Experiment 1. Data from six participants were excluded; three were excluded for incomplete data, and three were excluded because they exhibited a side bias (a preference for one side that was greater than two standard deviations away from the group mean). This left a final number of 67 participants, 55 females and 12 males. Participants in the Experimental Condition received reinforcement-predictive terminal stimuli whereas participants in the Control Condition received terminal stimuli that did not predict reinforcement. Within both the Experimental and Control Conditions there were three conditions which had terminal stimuli durations of 2, 8, and 20 s, respectively. Participants were randomly assigned to one of six conditions resulting in the following compositions: Experimental Condition 2 s (*n* = 14, 9 female and 5 male), Experimental Condition 8 s (*n* = 10, 7 female and 3 male), Experimental Condition 20 s (*n* = 9, 6 female and 3 male), Control Condition 2 s (*n* = 17, 11 female and 6 male), Control Condition 8 s (*n* = 9, 6 female and 3 male), and Control Condition 20 s (*n* = 8, 6 female and 2 male).

#### Apparatus

Experimental tasks were run using Windows 10 and presented on a ThinkVision L2250p 22in monitor with a resolution of 1,024 pixels × 768 pixels. All procedures were programed with OpenSesame version 3.2.8 (OpenSesame, RRID:SCR_002849, Mathôt et al., 2012). Participants experienced individual sessions in separate rooms with a researcher seated outside of that room in the waiting area of the lab.

Stimuli were presented 14.5 cm down the screen, and 10.5 cm across the screen from the left for the left location position or 38 cm across the screen from the left for the right position location. The square shape stimulus (2.61 cm × 3.16 cm) and the triangle shape stimulus (3.62 cm × 4.49 cm) appeared in either of these two locations. During forced trials, a gray circle (17.58 cm in circumference and 5.6 cm in diameter; rgb: 128, 128, and 128) was displayed in the side location that did not have a shape stimulus. Terminal stimuli appeared in the same side locations as the shape stimuli and measured 17.58 cm in circumference and 5.6 cm in diameter. The terminal stimuli displayed were colored either red (rgb: 255, 0, and 0), green (rgb: 0, 255, and 0), blue (rgb: 0, 0, and 255), or yellow (rgb: 255, 255, and 0). Reinforcement was an image of a gold coin (14.44 cm in circumference and 4.6 cm in diameter) that was centrally located 24 cm down the screen. The image of this coin was paired with an auditory stimulus similar to the sound of an old cash register (“*ka-ching!*”). In the top right corner of the screen (5.3 cm from the top of the screen and 34 cm from the left side of the screen) a green rectangle (0.89 cm × 3.52 cm) counted the reinforcers obtained. Directly above the counter were the words “Coins Received.”

#### Procedure

**FIGURE 2:**
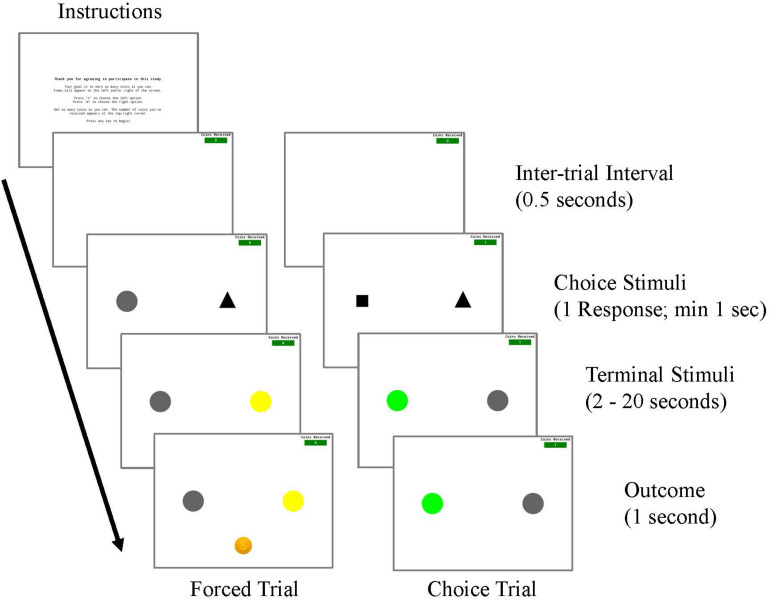
Procedure for Experiment 1.

The procedures used in Experiment 1, which were approved by the Institutional Review Board at Georgia Southern University, were similar to those previously used with non-human subjects (see Figure 1B) but modified for adult human participants (see Figure 2). An image of a coin and an auditory stimulus were used instead of food grain or pellets. Rather than responding on keys in an operant chamber, participants responded with the left and right arrow keys of a computer keyboard. Before the initiation of a session, participants were instructed to read along while a research assistant read the following directions on the computer screen aloud:

Thank you for agreeing to participate in this study.
Your goal is to earn as many coins as you can.
Items will appear on the left and/or right of the screen.
Press “LEFT ARROW” to choose the left option.
Press “RIGHT ARROW” to choose the right option.
Get as many coins as you can. The number of coins you’ve
received appears at the top-right corner.
Press any key to begin!

A session began once a key response was made. Forced trials were initiated by the presentation of either a square or a triangle choice stimulus in the left or the right side position. Following a single key response to the shape stimulus (FR1), the shape stimulus offset and a terminal color stimulus appeared in the same position. For Experimental Conditions, one choice stimulus was associated with two terminal stimuli, one of which was presented on 20% of the choices and was always followed by reinforcement, whereas the other was presented on 80% of these trials and was never followed by reinforcement. The other choice stimulus was associated with two terminal stimuli that occurred with equal frequency (50% of these choice trials), and were each followed by reinforcement 50% of the time. For Control Conditions, each choice stimulus was associated with two non-predictive terminal stimuli. The probability of appearance of these terminal stimuli was equated to those in the Experimental conditions. Specifically, for one choice stimulus, a terminal stimulus appeared on 20% of these shape trials while another terminal stimulus appeared on 80% of these choice trials and were each followed by reinforcement 20% of the time. For the other choice stimulus, both terminal links appeared with equal probability (50% of these choice trials) and were followed by reinforcement 50% of the time. The durations of the terminal stimuli were 2, 8, or 20 s, depending on the participant’s assigned condition. After the terminal stimulus duration elapsed a reinforcer was presented for 1 s according to the schedule, followed by a 0.5 s inter-trial interval. The inter-trial interval consisted of a blank screen with no stimuli present aside from the reinforcement counter. There were a total of 40 forced trials per session.

Choice trials were initiated with the simultaneous presentation of the shape stimuli in the left and right positions. A single key response to either shape stimulus was followed by the offset of both shape stimuli and the presentation of a terminal stimulus associated with the chosen shape. Probabilities associated with both terminal stimuli appearance and reinforcement were the same as in forced trials. There were a total of 20 choice trials per session.

A complete session consisted of 60 trials, with two blocks of 20 forced trials and 10 choice trials each. A greater number of forced trials were used to ensure that participants had ample experience with each alternative, and to replicate previous procedures that have also used more forced trials than choice trials (Stagner and Zentall, 2010; Zentall and Stagner, 2011). Trial presentation was randomized, but it was ensured that there would be one choice trial for every three trials and that there was never more than one choice trial presented consecutively. Both shape and terminal stimuli were counterbalanced for side presentation, and the shape stimuli that terminal stimuli were associated with were counterbalanced across conditions.

### Results

**FIGURE 3:**
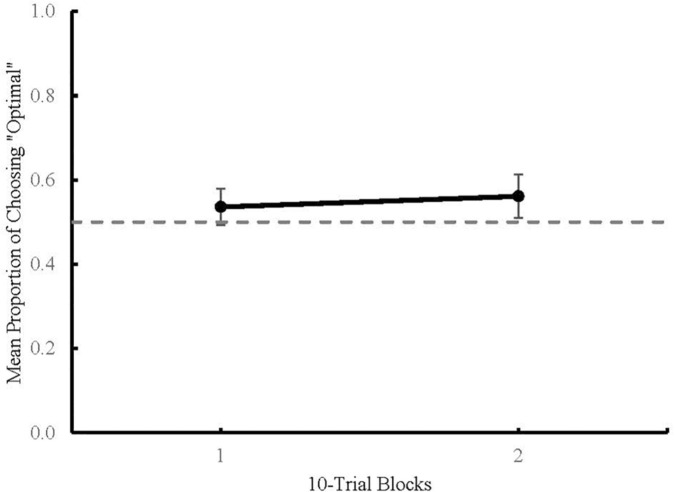
Mean proportion of optimal choices across 10-trial blocks in Experiment 1. Dashed line represents chance (0.5). Error bars represent 95% Confidence Intervals.

Statistical analyses were conducted using JASP 0.11.1 (JASP Team, 2019, RRID:SCR_015823). Means are reported with 95% confidence intervals and all significant effects are reported at a *p* < 0.05. Figure 3 shows the mean proportion of choosing the higher probability of reinforcement, or “optimal,” alternative plotted across blocks of 10 choice trials. A mixed Analysis of Variance (ANOVA) was used to determine the effects of Terminal Stimulus Type (predictive or non-predictive) and Terminal Stimulus Duration (2, 8, and 20 s) on mean proportion of optimal choices across Blocks 1 and 2. There was no main effect of Block [*F*(1) = 0.909, *p* = 0.344], no main effect of Terminal Stimulus Type [*F*(1) = 0.042, *p* = 0.839], and no main effect of Terminal Stimulus Duration [*F*(2) = 0.324, *p* = 0.725]. Due to the absence of any main effects, the data were collapsed across all conditions for further analysis. One sample two-tailed *t*-tests were run to detect any difference between chance performance (50%) and Block 1 [*t*(66) = 1.631, *p* = 0.108 (*M* = 0.54, 95% CI = ±0.04)], and between chance performance and Block 2 [*t*(66) = 2.312, *p* = 0.024 (*M* = 0.56, 95% CI = ±0.05)]. The *t-*test comparing Block 1 to chance was not significant but the *t-*test for Block 2 was, indicating that there was a change in participants’ choice behavior as a function of experience with the task.

### Discussion

In Experiment 1, participants allocated more choices to the optimal alternative that was followed by non-predictive stimuli during Block 2. This result indicates that participants developed a slight preference for the optimal alternative over the course of a session. This preference was not influenced by manipulations of Terminal Stimulus Type (non-predictive or predictive) or Terminal Stimulus Durations (2, 8, and 20 s) within this task.

Interestingly, not only did participants show no preference for predictive terminal stimuli, there was no clear indication that any of the terminal stimuli played a role in participants’ choice behavior. Considering this finding, it is possible that participants’ choice allocation in Experiment 1 was dependent on reinforcement history associated with each shape stimulus rather than the terminal stimuli that predicted reinforcement outcomes. Thus, if these terminal stimuli were removed all together, then it should not have an effect on how participants allocate their choices. To examine this, in Experiment 2, participants in the No Terminal Stimulus condition received the outcome of each trial directly after the shape stimulus/stimuli offset. The preference of participants in the No Terminal Stimulus condition was compared to the preference of new participants run in the Experimental and Control conditions. Additionally, because terminal stimulus duration did not play a role in participants’ choices in Experiment 1, the 2 s terminal stimulus duration was used in Experiment 2. This shortened the duration of each trial and allowed for more trials within a session, increasing the number of experiences that participants received with the contingencies in the task.

## Experiment 2

### Method

#### Participants

Participants (*N* = 47) were undergraduate students over the age of 18 and were recruited as described in Experiment 1. Participants signed a consent form before beginning an experimental session. Once the experimental session was completed, participants were debriefed. No deception was used in Experiment 2. Data from three participants were excluded; one was excluded for incomplete data, and two were excluded because they exhibited a side bias (using the same criteria as in Experiment 1). Thus, there was a final total of 44 participants, 29 females and 15 males. Participants were randomly assigned to either Experimental Condition 2 s (*n* = 11, 6 females and 5 males) or Control Condition 2 s (*n* = 10, 6 females and 4 males) utilized in Experiment 1, or the No Terminal Stimulus Condition (*n* = 23, 17 females and 6 males).

#### Apparatus

The apparatus and stimuli for Experiment 2 were identical to those in Experiment 1, save the exclusion of the “Coins Received” counter and the inclusion of a progress bar. A progress bar (13.5 cm in length) was located at the top of the computer screen (positioned 2 cm down the screen and 23.5 cm toward the middle of the screen). The shading on the progress bar increased every time the participant obtained a reinforcer, filling the bar through the task. Participants were informed that the progress bar filled as they grew closer to completing the task. Completion of the task was not contingent on the participant completely filling the progress bar.

#### Procedure

The procedures used in Experiment 2 were approved by the Institutional Review Board at Georgia Southern University. Before the initiation of a session, participants were instructed to read along while a research assistant read the following directions on the computer screen aloud:

Thank you for agreeing to participate in this study.
Your goal is to earn as many coins as you can.
Items will appear on the left and/or right of the screen.
Press “LEFT ARROW” to choose the left option.
Press “RIGHT ARROW” to choose the right option.
Earn as many coins as you can. Earning coins fills the Progress bar and moves you closer to completion.
Press any key to begin!

A session began once a key response was made. Contingencies for Experimental Condition 2 s and Control Condition 2 s were the same as in Experiment 1. For the No Terminal Stimulus Condition, trial outcomes were delivered immediately following the offset of a shape stimulus on forced trials or following the offset of both shape stimuli on choice trials. For the No Terminal Stimulus Condition, as was the case for both Experimental Condition 2 s and Control Condition 2 s (see Figure 1C), one shape stimulus produced reinforcement 20% of the time whereas the other shape produced reinforcement 50% of the time. There were 120 forced trials and 60 choice trials for all three conditions, for a total of 180 trials for a complete session. There were six total trial blocks consisting of 20 forced trials and 10 choice trials, and trial type presentation was randomized in the same manner as was used in Experiment 1. The progress bar was centered at the top of the screen and remained visible for the entirety of the experiment.

### Results

**FIGURE 4:**
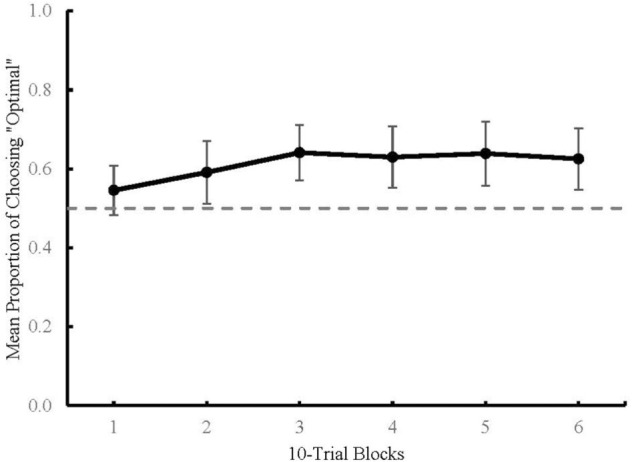
Mean proportion of optimal choices across 10-trial blocks in Experiment 2. Dashed line represents chance (0.5). Error bars represent 95% Confidence Intervals.

Figure 4 graphs the proportion of mean optimal responses across choice trials in Blocks 1 through 6, each consisting of 10 choice trials with 95% confidence intervals. An ANOVA was used to determine the effect of Terminal Stimulus Condition (predictive, non-predictive, or none) on the mean proportion of optimal choice across six blocks. Results revealed no main effect of block [*F*(5,205) = 2.086, *p* = 0.069]; the assumption of sphericity was violated and was adjusted with the Greenhouse–Geisser correction [*F*(3.69,151.38) = 2.086, *p* = 0.091]. There was also no main effect of condition observed [*F*(2,41) = 0.83, *p* = 0.443]. Due to the lack of main effects, the data were collapsed across conditions for further analysis. Table 1 shows results from one-sample *t*-tests which revealed that Blocks 2 through 6 differed significantly from chance (*M* = 0.591–0.641, 95% CI = ±0.07 – ±0.08). The significant *t*-tests for Blocks 2–6 indicate that participants’ choice behavior changed over the course of a session. Specifically, participants in all conditions preferred the optimal choice stimulus.

**TABLE 1:** Values for one sample *t*-tests from Experiment 2.

Experiment 2 one sample *T*-test comparison against chance					
Block	*t*-score	df	*p*-value	Mean	95% CI
1	1.431	43	0.16	0.545	[0.485, 0.605]
2	2.254	43	0.029*	0.591	[0.511, 0.671]
3	3.935	43	< 0.001*	0.641	[0.571, 0.711]
4	3.269	43	0.002*	0.63	[0.550, 0.710]
5	3.348	43	0.002*	0.639	[0.559, 0.719]
6	3.127	43	0.003*	0.625	[0.545, 0.705]

CI, confidence interval. All conditions were collapsed. Alternative hypothesis specifies the mean is different from chance (0.5). *p < 0.05.

### Discussion

Choice data from the Experimental and Control conditions in Experiment 2 show an absence of preference for predictive terminal stimuli. Further, participants in all the three conditions in Experiment 2 demonstrated a propensity to select the optimal shape stimulus. This preference was not affected by the type of terminal stimuli (or lack thereof) that were associated with the optimal shape stimulus. This finding is consistent with the conclusion from Experiment 1 that reinforcement history associated with the shape stimuli, rather than the predictive nature of the terminal stimuli, was the mechanism responsible for choice behavior in this task.

## General Results

The absence of an effect of Block on the group means indicating no significant change in choice allocation across a session could be interpreted as a lack of learning or preference acquisition. However, since reinforcers followed both alternatives, it was also possible that obtained reinforcement outcomes shaped individual participants’ choices across the duration of a session in both Experiments 1 and 2. That is, it is possible that selecting the suboptimal alternative was serendipitously reinforced, thereby increasing the likelihood of selecting it again. Specifically, the reinforcement outcomes following choice behavior may have been what determined future choices (Herrnstein, 1970). To determine whether obtained reinforcement might account for choice allocation, the generalized matching equation was applied (Baum, 1974) to the results of the first and last blocks of Experiments 1 and 2. The generalized matching equation is traditionally applied to choice data when the probabilities of reinforcement have been manipulated for an individual subject. However, Vollmer and Bourret (2000) applied the generalized matching equation to aggregated data, which enabled them to determine whether the choice between two alternatives collectively fit with predictions from obtained reinforcements.

To test the extent to which reinforcement history could account for participants’ choice behavior, we summed choice-allocation and obtained-reinforcement data within the First Block and Last Block for each participant. Then, the number of choices to the optimal-shape stimulus was divided by the number of choices to the suboptimal-shape stimulus (response ratio) and the number of reinforcements obtained following the optimal-shape stimulus was divided by the number of reinforcements obtained following the suboptimal-shape stimulus (reinforcement ratio). Data from participants that did not receive any reinforcers after choosing the suboptimal alternative were excluded from these analyses, resulting in the exclusion of six participants from the First Block and one participant from the Last Block in Experiment 1, and two participants from the First Block and three participants from the Last Block of Experiment 2.

**FIGURE 5:**
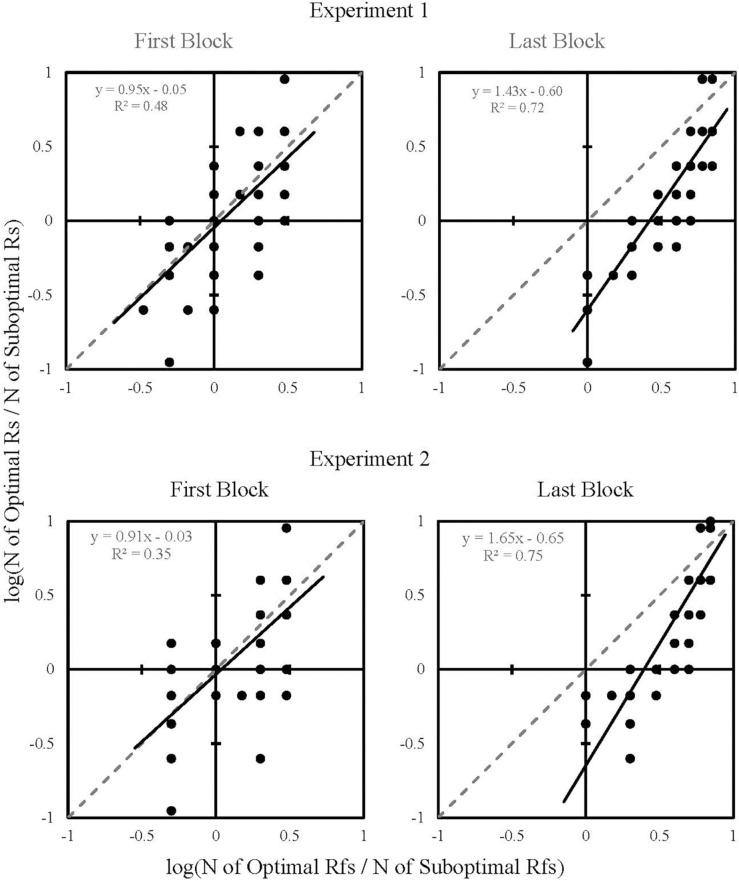
Log response ratios plotted as a function of log reinforcement ratios for First Block and Last Block of Experiments 1 and 2. Each dot represents one participant. The regression line equation and corresponding *R*^2^ values are in the upper-left quadrant of each plot.

Figure 5 shows each participant’s response and reinforcement ratios as one data point for the First and Last Blocks of Experiments 1 and 2. Response and reinforcement ratios were logged (base 10) to allow linear regression analysis. The data were fitted to regression lines, the equations of which appear in the upper-left quadrants of each plot. Response ratios significantly correlated with reinforcement ratios in Experiment 1 [First Block, *r*(59) = 0.69, *p* < 0.001; Last Block, *r*(64) = 0.85, *p* < 0.001] and Experiment 2 [First Block, *r*(40) = 0.59, *p* < 0.001; Last Block, *r*(39) = 0.87, *p* < 0.001]. Referring to goodness of fit, it appears that obtained reinforcement became a better predictor of response allocation from First Block [Experiment 1: *R*^2^ = 0.48 (RMSE = 0.26); Experiment 2: *R*^2^ = 0.35 (RMSE = 0.33)] to Last Block [Experiment 1: *R*^2^ = 0.72 (RMSE = 0.23); Experiment 2: *R*^2^ = 0.75 (RMSE = 0.25)], providing further evidence that the participant’s choices were influenced by obtained reinforcers, not by the predictiveness of the terminal stimuli.

## General Discussion

The results obtained from Experiments 1 and 2 contribute to the expansion of literature on adult human choice behavior and how it compares to that of non-humans. Perhaps most interesting is that reinforcement predictors seemed to play no role in adult human choice behavior within the scope of this task, which is different than what has been found in pigeons (Stagner and Zentall, 2010). Similar results were found recently with adult human participants in that they also did not show any preference for reinforcement predictors (McDevitt et al., 2019). A closer look at the present results illuminates that adult human choice in this task was driven by reinforcement history rather than reinforcement predictors. Additionally, removing predictive stimuli from the task all together in Experiment 2 had no effect on participants’ choice behavior. These findings suggest that reinforcement predictors within this two-alternative task did not play a role in participants’ choice allocation.

When comparing the absence of a preference for reinforcement predictors from adult humans in the present studies as well as McDevitt et al. (2019) to that of developmentally delayed children, a clear difference can be observed in choice behavior. That is, adult humans do not show a preference for predictive stimuli (McDevitt et al., 2019) but, under certain conditions, developmentally delayed children do (Lalli et al., 2000). When given a similar two-alternative task, rhesus macaques (Smith et al., 2017) showed a similar preference to that of developmentally delayed children (Lalli et al., 2000). From consideration of these findings from higher-order primates, a quantitative difference in preference for reinforcement predictors emerges across adult humans, developmentally delayed children, and rhesus macaques.

Previous observations from rats suggest that they may choose a suboptimal alternative that provides reinforcement predictors under certain conditions (Chow et al., 2017; Cunningham and Shahan, 2019). However, they do not do so, at least certainly not to the extent to which pigeons do, when given the equivalent task that has been used when observing this behavior in pigeons (Stagner and Zentall, 2010; Zentall and Stagner, 2011). More recently, pigeons, rats, and dogs were presented with a two-alternative task in which the two alternatives paid out equally but one produced predictive terminal stimuli (Jackson et al., 2020), much like the procedure used by Roper and Zentall (1999). Just as Roper and Zentall (1999) found, pigeons showed a preference for the alternative that produced predictive stimuli. Interestingly, dogs and rats showed no such preference (Jackson et al., 2020). The absence of a preference for predictive stimuli from dogs and sometimes from rats (Jackson et al., 2020) is similar to the present studies’ findings as well as those from McDevitt et al. (2019) with human participants.

Taken together, previous findings as well as the present results help to illuminate the value of reinforcement predictors within a two-alternative choice task. Adult human participants (McDevitt et al., 2019) and dogs (Jackson et al., 2020) do not seem to prefer predictive stimuli over non-predictive stimuli. Human children with developmental delays (Lalli et al., 2000), rhesus macaques (Smith et al., 2017), and rats (Chow et al., 2017; Cunningham and Shahan, 2019) show preference for predictive stimuli under certain contextual variables. Pigeons show a strong and clear preference for predictive stimuli (Roper and Zentall, 1999; Stagner and Zentall, 2010; Zentall and Stagner, 2011; McDevitt et al., 2019; Jackson et al., 2020). All of these studies contribute evidence that suggests that there are differences across vertebrate species with respect to preference (or lack thereof) for reinforcement predictors, which is in contrast to Macphail (1985) hypothesis. The greatest disparity is perhaps between the strong preference that pigeons (Stagner and Zentall, 2010; Zentall and Stagner, 2011) show for predictive stimuli and the lack of such a preference observed from adult humans (McDevitt et al., 2019).

The present studies observed adult human preferences for predictive stimuli using a two-alternative task. This task was very similar in format to what pigeons have received in past research (Stagner and Zentall, 2010; Zentall and Stagner, 2011; Stagner et al., 2012; Laude et al., 2014). Adult human participants, unlike pigeons, did not show a preference for stimuli that predict reinforcement. This finding suggests a possible qualitative difference between pigeons and adult humans within this type of two-alternative task. Differences between the two species have also been observed when measuring preferences for stimuli that look like they would produce reinforcement. These stimuli can be thought of as “near hits” in that visually they look very similar to stimuli that are predictive of a win. Slot machines, for example, produce some turns (or trials) that may visually look more like a win than a loss. For example, if there are three reels in a slot machine, two of the three reels would stop on matching stimuli during a “near-hit” trial. This might visually appear closer to a win than if all three reels produce different stimuli—a clear loss. Although these “near-hit” trials are equivalent to clear losses, adult human participants show a preference for slot machines that produce these trials more frequently under conditions in which they have experienced losses (MacLin et al., 2007).

To test for this preference in pigeons, Stagner et al. (2015) gave pigeons a two-alternative task. Choice of one alternative sometimes yielded “near-hit” trials in which a positive stimulus that signaled food would appear but then would change to a negative stimulus signaling the absence of food. Choice of the other alternative resulted in the same amount of food overall, but did not produce “near-hit” trials. Pigeons did not prefer the alternative that produced “near-hit” trials (Stagner et al., 2015) which is different from what was found with human participants (MacLin et al., 2007). Two-alternative tasks like the one used by Stagner et al. (2015) have been suggested to be analogous to human gambling procedures. However, when considering the differences between pigeons and adult human participants on tasks such as this, it must be noted that perhaps this task does not produce behavior that is analogous to human gambling or that it only does so under specific conditions with specific subjects. When considering Macphail (1985) proposal that there should not be differences between vertebrates, the findings from pigeons and humans with respect to preferences for predictive stimuli are in sharp contrast. When considering the present findings, adult human choice allocation seems more driven by reinforcement history. Conversely, the predictive nature of stimuli seems to be valuable for pigeons at the expense of how often food is actually presented.

Some evidence for preference for predictive stimuli rather than overall amount of reinforcement by pigeons has been observed in the same type of two-alternative task (Stagner et al., 2012). When both alternatives sometimes produced a terminal stimulus that predicted reinforcement 100% of the time, pigeons were indifferent between the two alternatives. What is interesting about this is that while both alternatives were associated with a stimulus that predicted reinforcement, a reinforcement predictor occurred more frequently following choice of one alternative (50% of the time) than following choice of the other alternative (20% of the time). Similar results were also found by Zentall et al. (2015) in that pigeons showed preference for the reliability of a reinforcement predictor independent of its frequency. Considering the findings from Stagner et al. (2012) and Zentall et al. (2015), pigeons appeared to be selecting the alternative that produced the best stimulus predictive of reinforcement within the tasks. However, if pigeons’ choice within these tasks was determined by overall reinforcement associated with each alternative, then there would have been more choice allocation to the optimal alternative that provided more reinforcement. The present findings suggest that adult human participants might take a more global view when allocating choices, which is in contrast to the findings from pigeons (Stagner et al., 2012; Zentall et al., 2015).

In addition, future research with adult human participants focusing on the effects of deprivation and depletion may also provide further insight into predictive stimuli preferences. Motivating operations could be explored by systematically manipulating contextual variables, such as food deprivation and social enrichment. Both have been found to have an effect on pigeons’ preference for predictive stimuli. Specifically, more food-deprived pigeons chose an alternative that produced predictive stimuli but less reinforcement over time whereas less food-deprived pigeons chose optimally (Laude et al., 2012). Additionally, pigeons that received social enrichment were much slower to show a preference for a predictive suboptimal alternative than were their control counterparts that received no such social enrichment (Pattison et al., 2013). The performance by less food-deprived pigeons (Laude et al., 2012), and early performance by socially enriched pigeons (Pattison et al., 2013), more closely resemble the data that has been collected using adult human participants in the present studies and from McDevitt et al. (2019). In the future, motivating operations could be examined in adult humans to observe if similar results are found.

Considering the present results, pigeons appear to perform differently than rats (Chow et al., 2017; Cunningham and Shahan, 2019), dogs (Jackson et al., 2020), rhesus macaques (Smith et al., 2017), developmentally delayed children (Lalli et al., 2000), and especially adult humans (McDevitt et al., 2019) within this task. When considering the performance of adult humans and pigeons specifically, the extant difference in preference for reinforcement-predictive stimuli could indicate that the two species are fundamentally different. This pervasive finding is in contradiction to the notion that vertebrate species should not differ (Macphail, 1985), and suggests that further examination into factors which account for the choice-allocation differences across vertebrate species is warranted.

## Data Availability Statement

The datasets generated for this study are available on request to the corresponding author.

## Ethics Statement

The studies involving human participants were reviewed and approved by Institutional Review Board Georgia Southern University. The patients/participants provided their written informed consent to participate in this study.

## Author Contributions

JS, KB, SB, VE, JJ, and RS were part of the development of all procedures used in Experiments 1 and 2. KB, SB, VE, JJ, and RS ran statistical analyses and constructed figures used. KB programed procedures for Experiments 1 and 2 using OpenSesame. SB, VE, JJ, and RS collected the data. JS wrote this manuscript. All authors contributed to the article and approved the submitted version.

## Conflict of Interest

The authors declare that the research was conducted in the absence of any commercial or financial relationships that could be construed as a potential conflict of interest.

## Footnotes

1 www.sona-systems.com
